# Platelet-Rich Plasma Improves Pregnancy Outcomes in Moderate to Severe Intrauterine Adhesion: A Retrospective Cohort Study

**DOI:** 10.3390/jcm12041319

**Published:** 2023-02-07

**Authors:** Daner Qiu, Xifeng Xiao, Wenting Wang, Wanlin Zhang, Xiaohong Wang

**Affiliations:** Reproductive Medical Center, Department of Obstetrics and Gynecology, Tangdu Hospital, Air Force Military Medical University, Xi’an 710032, China

**Keywords:** Asherman’s syndrome, intrauterine adhesion, platelet-rich plasma, hysteroscopic adhesiolysis, propensity score matching

## Abstract

The purpose of the present study was to investigate the therapeutic effects of platelet-rich plasma (PRP) in women with moderate to severe intrauterine adhesion (IUA). A retrospective cohort study was conducted at a reproductive medical center between July 2020 and June 2021 to compare the clinical pregnancy rate of two groups (PRP and non-PRP groups) after hysteroscopic adhesiolysis. A multivariate logistic regression analysis and propensity score matching (PSM) were performed to minimize potential bias. According to our inclusion and exclusion criteria, 133 patients were finally enrolled and divided into the PRP group (*n* = 48) and non-PRP group (*n* = 85). In the primary comparison, the clinical pregnancy rate in the PRP group was higher than that in the non-PRP group (41.7% vs. 28.2%, *p* = 0.114), albeit without statistical significance. Multivariate logistic regression analysis was performed, and the results of the adjusted model showed that PRP treatment significantly improved the clinical pregnancy rate (adjusted OR = 3.00, 95% CI = 1.22–7.38, *p* = 0.017). After PSM, the clinical pregnancy rate was higher in the PRP group than that in the non-PRP group (46.2% vs. 20.5%, *p* = 0.031). Based on the present study, we concluded that intrauterine perfusion of PRP had great potential in improving the clinical pregnancy rate in patients with moderate to severe IUA. Therefore, we recommend the application of PRP in the treatment of IUA.

## 1. Introduction

Intrauterine adhesion (IUA) is the secondary endometrial injury disease [1] that leads to amenorrhea, infertility, and poor pregnancy outcomes in assisted reproductive technology (ART) in women of childbearing age. The main etiology of IUA is pregnancy-related curettage, such as post-abortion, miscarriage, and postpartum curettage [2,3,4]. These procedures may result in the endometrial basal layer being too impaired to completely repair or restore the endometrium. Instead, fibrous and fibro-muscular adhesive bands between the inner wall of the uterine cavity formed during endometrial repair. With adhesions advancing, the uterine cavity becomes smaller and obstructed, and its structure becomes distorted. At present, comprehensive treatment of IUA not only focuses on the removal of adhesive bands and restoration of the shape of the uterine cavity, but also attaches importance to the repair and regeneration of the impaired endometrium. An appropriate endometrial status is vital for embryo implantation. However, most patients with moderate to severe IUA have too thin or inferior endometrium to support a pregnancy after surgery.

Platelet-rich plasma (PRP) is the highly concentrated platelet plasma obtained from autologous peripheral venous blood of patients [5,6]. The activation of α particles of platelets can release cytokines and growth factors, and form an appropriate biological microenvironment in the endometrium, which is conducive to the repair of damaged tissues [7]. However, its application in the treatment of IUA is still in the preliminary stages, and the effects of PRP as an adjuvant therapy after hysteroscopic adhesiolysis remains inconclusive. Herein, a retrospective cohort study was conducted to investigate the therapeutic effects of PRP in women with moderate to severe IUA, which we hope will provide evidence for the application of PRP in the treatment of IUA.

## 2. Materials and Methods

### 2.1. Study Design

All clinical records from the electronic medical database of a tertiary reproductive medical center from July 2020 to June 2021 were retrospectively collected. A total of 176 women who underwent hysteroscopic adhesiolysis at our center were eligible for inclusion in this study. Of these, 43 patients who met the following exclusion criteria were excluded: (1) incomplete medical records; (2) unicornuate or bicornuate uterus; (3) polycystic ovarian syndrome; and (4) history of tuberculosis (TB). Finally, 133 patients were included in the analysis. Before surgery, the option of intrauterine infusion of PRP as one of the adjuvant treatments would be informed in detail to all patients who planned to receive hysteroscopic adhesiolysis. Among the 133 patients, 85 patients who did not receive PRP treatment were included in the control group, whereas 48 who underwent intrauterine perfusion of PRP were included in the PRP group.

Baseline demographic characteristics included age, body mass index (BMI), preoperative estradiol level, daily dose of postoperative estradiol, adhesion grades (according to the scoring system proposed by the American Fertility Society [8], Table 1), history of hysteroscopic surgery, type of previous hysteroscopic surgery, the number of curettages in previous, menstrual cycle (menstrual cycle of 28 ± 7 days was defined as regular; otherwise, irregular), and method of conception. The detailed information is listed in Table 2.

### 2.2. Ethical Statement

This study was approved by the Medical Ethics Committee of Tangdu Hospital, Air Force Military Medical University (No. TDLL-202202-05). The data were anonymized, and the requirement for informed consent was waived.

### 2.3. Hysteroscopic Adhesiolysis Procedure

The cervix was dilated using a gelatin stick one day before surgery. All procedures were performed under para-uterine infiltration anesthesia (lidocaine combined with dezocine). The dilating stick was withdrawn at the beginning of the surgery, and the hysteroscope was placed in the cavity to inspect its overall condition. During surgery, 0.9% normal saline was used as the dilatation medium. The hard or dense fibro-muscular adhesive bands were separated using bipolar electric needles (Storz, Tuttlingen, Germany) under hysteroscopic surveillance. When all adhesive bands were separated and the shape of the uterine cavity returned to normal, the hysteroscopic instruments were slowly withdrawn.

### 2.4. PRP Preparation and the Perfusion Scheme

PRP was prepared manually in a test tube using a two-step centrifugal process in some published studies [9,10]. In the present study, we prepared PRP using a commercially available automated device. This preparation method has the following characteristics: the preparation process is automatic and conveniently; approximately 30 mL PRP can be prepared by extracorporeal circulation of 400 mL whole blood for later use (the volume of isolated whole blood extracted from patients’ peripheral veins varied with the platelet concentration and the volume of PRP); and the concentrate of platelets is relatively higher and remains steady [11,12].

The detailed process is concluded below. PRP was isolated automatically from the whole blood by using an automatic blood component separator (NGLXCF3000; Nigale Biomedical Co., Ltd., Sichuan, China). The operator entered relevant information into the machine, including patient’s height, body weight, gender, red blood cell specific volume, and platelets. In addition, the collection parameters were adjusted, centrifuged at 5500 r/min, the collection and back-transfusion rate were set as 20–100 mL/min and 20–120 mL/min, respectively, and the ratio of anticoagulant to whole blood was 1:10. After 30 mL of PRP was automatically isolated by the kit, the remaining platelets, red blood cells, white blood cells, and the plasma were transfused back into the patients. The prepared PRP was divided into 5 packets and cryopreserved at −80 °C until use. According to the data provided by the manufacturer, the platelet concentration ranges of PRP are 1180.08 ± 301.21× 103/µL, and the concentration ranges of white blood cells are 35.14 ± 20.98 ×10/µL. In the PRP perfusion scheme, clinicians injected 5–6 mL of PRP into the uterine cavity through a Foley catheter on the 1st, 3rd, and 5th day after surgery, and the 1st and 3rd day after the next menstruation ceased.

### 2.5. Standard Cares

In addition to PRP treatment, the following adjuvant therapies were performed routinely in our center. A Foley catheter was placed in the uterine cavity, and 5 mL of hyaluronic acid gel (YiShuKang^®^; Materia Medica Co., Changzhou, China) was injected through the Foley catheter at the end of surgery. The Foley catheter was retained in the uterine cavity as a structural support for 7 days. Thereafter, the following interventions were performed: (1) antibiotic administration with cefazoline sodium 1 g, twice daily for 7 days and ornidazole 500 mg, twice daily for 7 days; (2) hormone replacement therapy with estradiol valerate (Progynova, Bayer Healthcare Co., Ltd., Guangzhou branch) 0–4 mg/d for 17–21 days combined with progesterone (Dydrogesterone, Abbott Biologicals B.V., The Netherlands) 20 mg/day for the last 10 days of the cycle; and (3) an outpatient hysteroscopy examination was performed 7 days after surgery. The next examination time point was determined by the uterine recovery condition, which was recommended by experienced clinicians. During the examination, if re-adhesion occurs, the loose or flimsy adhesive bands would be separated bluntly by clinicians. The outpatient hysteroscopic review would stop when the uterine cavity has returned to a state that can support pregnancy or when the physician considers that secondary surgery is necessary.

### 2.6. Clinical Outcomes

The primary study endpoint was the clinical pregnancy rate, which was defined as an intrauterine gestational sac with heartbeats detected by ultrasound at the end of the 9-month follow-up period after surgery. The secondary outcome was the improvement of menstrual volume 3-months after surgery, which was recorded as either improved or not improved based on the subjective feelings of the patients.

### 2.7. Statistical Analysis

Continuous variables are expressed as mean ± standard deviation or median (25th, 75th percentile), and categorical variables are expressed as percentages. Comparisons of the 2 groups were performed using Chi-square tests (for categorical variables), Fisher’s exact test (the theoretical number of the categorical variables < 10), Student’s test (normally distributed continuous variables), or Mann–Whitney U test (abnormally distributed continuous variables).

In retrospective studies, non-randomized allocation and unbalanced baseline characteristics may affect the primary comparison of the 2 groups and undermine the reliability of the results. Hence, we used the multivariate logistic regression analysis to assess the association between the PRP perfusion and reproductive outcome. In the adjusted model, we adjusted for age, adhesion grades, and history of hysteroscopic surgery as potential confounding factors based on clinical experience and published studies.

Propensity score matching (PSM) has attracted increasing interest for avoiding selection bias and increasing the level of evidence. To further confirm our results, we calculated the propensity scores by logistic regression analysis including BMI, age, adhesion grades, history of hysteroscopic surgery, and method of conception. These variables were selected according to clinical practice experience and the unbalanced variables presented in Table 2. We matched patients by propensity score using the nearest-neighbor method with a matching ratio of 1:1. In addition, the stratified multivariate logistic regression by age, adhesion grades, and history of hysteroscopic surgery was conducted.

All statistical analyses were performed using statistical packages R (The R Foundation; http://www.r-project.org, accessed on 1 January 2023; version 4.2.0) and EmpowerStats (www.empowerstats.net, accessed on 1 January 2023; X&Y solutions, Inc., Boston, MA, USA).

## 3. Results

### 3.1. Baseline Characteristics of PRP and Non-PRP Groups

As shown in Table 2, there were no significant differences between the two groups in terms of age, BMI, preoperative estradiol level, daily dose of postoperative estradiol, adhesion grades, number of curettages, menstrual cycle, and method of conception. However, more patients in the PRP group had a history of hysteroscopic surgery. In the PRP group, 22 (45.8%) patients had a history of hysteroscopic surgery, among which 18 patients had a history of hysteroscopic adhesiolysis. In the non-PRP group, 16 (18.8%) patients had a history of hysteroscopic surgery, among which 11 patients had a history of hysteroscopic adhesiolysis. In the primary comparison, the clinical pregnancy rate in the PRP group was higher than that in the non-PRP group (41.7% vs. 28.2%, *p* = 0.114); however, the difference between the two groups was not statistically significant. In the non-PRP group, 24 patients had a clinical pregnancy, among which 16 (66.7%) patients became pregnancy by ART, and 8 (33.3%) patients became pregnant through natural intercourse. In the PRP group, 20 patients had a clinical pregnancy, among which 17 (85.0%) patients became pregnant by ART, and 3 (15.0%) patients became pregnant through natural intercourse.

### 3.2. The results of Multivariate Regression Analysis in Different Models

In the multivariate logistic regression analysis, the results of the crude model showed a slight improvement in the clinical pregnancy rate in the PRP group; however, the difference was not statistically significant (OR = 1.82, 95% CI = 0.86–3.82, *p* = 0.116). In the adjusted model, we adjusted for age, adhesion grades, and history of hysteroscopic surgery as potential confounding factors, and the results showed that PRP treatment can significantly improve the clinical pregnancy rate of patients with moderate to severe IUA (adjusted OR = 3.00, 95% CI = 1.22–7.38, *p* = 0.017). The detailed results are shown in Table 3.

### 3.3. The Results of PSM

According to the PSM method, the patients in the PRP group were matched with 85 patients in the non-PRP group at a ratio of 1:1 to balance the baseline clinical characteristics and minimize the selection bias of two groups. After PSM, 78 patients (39/group), were enrolled in the study for further analysis. The detailed characteristics of each matched pair are listed in Table 4. The clinical pregnancy rate was higher in the PRP group (46.2% vs. 20.5%, *p* = 0.031).

### 3.4. The Results of Stratified Analysis

As shown in Table 5, we also investigated the relationship between the PRP perfusion treatment and the reproductive outcome in stratified groups. All patients were stratified by age, adhesion grades, and history of hysteroscopic surgery. In the stratified analysis by age, the PRP treatment significantly improved the clinical pregnancy rate of patients in the <35 years groups (OR = 3.02, 95% CI = 1.16–7.90, *p* = 0.024). Similarly, PRP perfusion therapy improved the reproductive outcome in IUA patients diagnosed with moderate IUA or without a history of hysteroscopic surgery (OR = 2.73, 95% CI = 1.06–7.02, *p* = 0.037), (OR = 2.67, 95% CI = 1.06–6.73, *p* = 0.038).

## 4. Discussion

Autologous PRP has several advantages, such as convenience and ease of preparation as well as no immunogenicity [13]; therefore, it has great potential for clinical applications. Currently, it is widely used in orthopedics, plastic and maxillofacial surgery, sports medicine, refractory wounds, and other fields where tissue regeneration is required [13]. PRP application in impaired endometrial repair mainly depends on the high concentration of growth factors, cytokines, and anti-inflammatory factors released by the α granules of platelets. Studies have shown that the concentration of PDGF, TGF-β, VEGF, and EGF in PRP is three to seven times that of regular serum [14,15]. In addition, the proportion of each factor is similar to the normal status of injured tissue repair. Each type of growth factor plays a role in promoting cell proliferation, migration, angiogenesis, and anti-inflammatory activity [16,17,18,19], which accelerates the process of repair of the injured endometrium [20,21].

Some studies have evaluated the effects of autologous PRP in the inferior endometrium, and the results have shown that PRP treatment results in a striking improvement in the pregnancy outcome of frozen embryo transfer cycles and the blood flow state of the endometrium [22,23,24]. In recent years, PRP was initially applied in the repair of a damaged endometrium associated with IUA, and positive effects have been observed.

In 2015, a research team from the Sixth Affiliated Hospital of Sun Yat Sen University of China first reported that autologous PRP had positive effects on promoting endometrial repair and improving reproductive prognosis in five infertility patients with a thin endometrium. One of the five patients was diagnosed with IUA, and hysteroscopy after surgery showed that the endometrium could not completely cover the wound surface. However, 48–72 h after receiving PRP perfusion, the endometrial thickness increased, and a frozen embryo was successfully transferred [9]. Aghajanova et al. [25] subsequently published a series of case reports in which two patients with IUA received PRP perfusion therapy after surgery, and all became pregnant. Another study that included 60 patients with primary or secondary infertility and severe IUA evaluated the effects of PRP gel infusion after hysteroscopic adhesiolysis. Thirty patients received PRP gel plus a Foley catheter, while the control group received a Foley catheter only. The results showed that the postoperative menstrual duration in the PRP group was significantly longer than that in the control group [26].

Peng et al. [27]. conducted a retrospective cohort study to explore the effectiveness of PRP by comparing the IUA scores and postoperative chemical pregnancy rates of the PRP and balloon groups after surgery. The results showed that the chemical pregnancy rate in the PRP group was higher than that in the balloon group, but the difference was not statistically significant. Similarly, a small-sample randomized controlled trial in 2020 showed that assisted intrauterine PRP perfusion after surgery could not significantly improve menstruation, and there was no statistically significant difference in the postoperative score of IUA compared with the control group [15]. These results indicate that postoperative intrauterine infusion of PRP may be a potential treatment method for IUA. Rigorously designed studies with large sample sizes are needed to further confirm the therapeutic effects of PRP in IUA treatment.

Nevertheless, based on the small sample size and non-randomized studies, evidence related to the effect of PRP in IUA is insufficient. In the present study, we conducted a retrospective cohort study with a larger sample size to investigate the therapeutic effects of PRP on improving pregnancy outcomes. Considering the unbalanced index between the two groups, we used PSM to try to obtain balanced groups and minimize potential bias for a more reliable result. After PSM, there was a significant difference in the clinical pregnancy rates between the two groups (46.2% vs. 20.5%, *p* = 0.031). In addition, the stratified analysis revealed the target population, which could receive more benefits from PRP perfusion therapy.

So far, the perfusion volume and the times of perfusion are inconsistent. The perfusion volume varied from 0.5 mL to 4 mL [9,10,28,29], and the times of perfusion varied from one to three times [14,15,28,29]. In the present study, we injected PRP on the first, third, and fifth day after surgery, and the first and third day after the next menstruation ceased. This perfusion scheme can increase the local concentration and prolong the functional time, which might improve the therapeutic effect of PRP in IUA treatment. Our results add to the evidence of PRP in clinical use and IUA treatment. Our study has some limitations. First, this is a retrospective study, and there are some intrinsic limitations that could not be completely avoided, such as selection bias [30], although we used PSM to minimize such bias. Second, the sample size after PSM is relatively small, which might compromise the statistical power. Well-designed, randomized controlled studies are required in the future. Third, our study was conducted in a single center in northwest China; hence, caution should be exercised when extrapolating the conclusions to other populations. A multi-center study is required in the future.

## 5. Conclusions

Based on the present cohort study, we concluded that intrauterine perfusion of PRP after hysteroscopic adhesiolysis can improve the clinical pregnancy rate of patients with moderate to severe IUA. PRP seems to have great potential in the treatment of IUA. More studies are needed to further evaluate this therapy.

## Figures and Tables

**Table 1:** The American Fertility Society classification of IUA, 1988 [8].

Extent of cavity involved	<1/3	1/3–2/3	>2/3
Score	1	2	4
Type of adhesion	Filmy	Filmy and Dense	Dense
Score	1	2	4
Menstrual pattern	Normal	Hypomenorrhoea	Amenorrhoea
Score	0	2	4
Prognostic classification	Hysteroscopy score (total)		
Stage I (Mild)	1–4		
Stage II (Moderate)	5–8		
Stage III (Severe)	9–12		

**Table 2:** Characteristics of the study population.

Variables	Non-PRP Group (N = 85)	PRP Group (N = 48)	*p*
BMI (kg/m^2^)	22.6 ± 3.6	23.2 ± 3.5	0.380
Preoperative estradiol level (pg/mL)	77.1 (58.9–123.8)	85.6 (52.7–181.9)	0.942
Age (years)	34.9 ± 4.4	34.4 ± 4.8	0.579
Daily dose of postoperative estradiol (mg)	3.4 ± 1.0	3.7 ± 0.9	0.105
Age			0.566
<35 years	47 (55.3%)	29 (60.4%)	
≥35 years	38 (44.7%)	19 (39.6%)	
Adhesion grades			0.057
Moderate	60 (70.6%)	26 (54.2%)	
Severe	25 (29.4%)	22 (45.8%)	
History of hysteroscopic surgery			<0.001
No	69 (81.2%)	26 (54.2%)	
Yes	16 (18.8%)	22 (45.8%)	
Type of previous hysteroscopic surgery			0.481
Hysteroscopic adhesiolysis	11 (68.8%)	18 (81.8%)	
Separation of uterine septum	4 (25.0%)	2 (9.1%)	
Electroresection of endometrial polyps	0 (0.0%)	1 (4.5%)	
Two or more	1 (6.2%)	1 (4.5%)	
Number of curettage in previous			0.329
None	12 (14.1%)	10 (20.8%)	
Once	35 (41.2%)	20 (41.7%)	
Twice	24 (28.2%)	15 (31.2%)	
Three or more times	14 (16.5%)	3 (6.2%)	
Menstrual cycle			0.124
Regular	72 (84.7%)	45 (93.8%)	
Irregular	13 (15.3%)	3 (6.2%)	
Method of conception			0.137
Spontaneous conception	32 (37.6%)	12 (25.0%)	
Assisted reproductive technology	53 (62.4%)	36 (75.0%)	
**Clinical outcomes**			
The improvement of menstrual volume			0.824
Not improved	25 (29.4%)	15 (31.2%)	
Improved	60 (70.6%)	33 (68.8%)	
Pregnancy outcome within 9 months follow-up			0.114
Not yet pregnant	61 (71.8%)	28 (58.3%)	
Clinical pregnancy	24 (28.2%)	20 (41.7%)	

**Table 3:** The relationship between intrauterine perfusion of PRP and pregnancy outcomes in different models.

Exposure	Non-Adjusted *		Adjusted **	
Perfusion with PRP	OR (95% CI)	P	OR (95% CI)	P
No	1.0		1.0	
Yes	1.82 (0.86, 3.82)	0.116	3.00 (1.22, 7.38)	0.017

* Non-adjusted model adjusted for: none. ** Adjusted model adjusted for: age, adhesions grades, and history of hysteroscopic surgery.

**Table 4:** Characteristics of propensity-matched groups.

Variables	Non-PRP Group (N = 39)	PRP Group (N = 39)	Standardized Difference	*p*
BMI (kg/m^2^)	22.7 ± 3.5	23.4 ± 3.4	0.203	0.373
Age			0.052	1.000
<35 years	22 (56.4%)	23 (59.0%)		
≥35 years	17 (43.6%)	16 (41.0%)		
Adhesion grades			0.104	0.819
Moderate	23 (59.0%)	21 (53.8%)		
Severe	16 (41.0%)	18 (46.2%)		
History of hysteroscopic surgery			0.000	1.000
No	25 (64.1%)	25 (64.1%)		
Yes	14 (35.9%)	14 (35.9%)		
Method of conception			0.109	0.810
Spontaneous conception	14 (35.9%)	12 (30.8%)		
Assisted reproductive technology	25 (64.1%)	27 (69.2%)		
**Clinical outcomes**				
The improvement of menstrual volume			0.111	0.806
Not improved	11 (28.2%)	13 (33.3%)		
Improved	28 (71.8%)	26 (66.7%)		
Pregnancy outcome within 9 months follow-up			0.565	0.031
Not yet pregnant	31 (79.5%)	21 (53.8%)		
Clinical pregnancy	8 (20.5%)	18 (46.2%)		

**Table 5:** The results of stratified analysis.

Variables	OR (95% CI)	*p*
Age		
<35 years	3.02 (1.16, 7.90)	0.024
≥35 years	0.60 (0.14, 2.56)	0.493
Adhesion grades		
Moderate	2.73 (1.06, 7.02)	0.037
Severe	1.54 (0.36, 6.66)	0.560
History of hysteroscopic surgery		
No	2.67 (1.06, 6.73)	0.038
Yes	1.63 (0.34, 7.79)	0.544

## Data Availability

The data and material underlying this article will be shared on reasonable request to the corresponding author.

## References

[1] Asherman J.G. (1948). Amenorrhoea traumatica (atretica). J. Obstet. Gynaecol. Br. Emp..

[2] Zhao X., Sun D., Zhang A., Huang H., Zhu X., Yi S., Xu D. (2022). Uterine Cavity Parameters Evaluated by Hysteroscopy can Predict the Live Birth Rate for Intrauterine Adhesion Patients. Front. Med..

[3] Khan Z., Goldberg J.M. (2018). Hysteroscopic Management of Asherman’s Syndrome. J. Minim. Invasive Gynecol..

[4] Deans R., Vancaillie T., Ledger W., Liu J., Abbott J.A. (2018). Live birth rate and obstetric complications following the hysteroscopic management of intrauterine adhesions including Asherman syndrome. Hum. Reprod..

[5] Amable P.R., Carias R.B., Teixeira M.V., da Cruz Pacheco I., Corrêa do Amaral R.J., Granjeiro J.M., Borojevic R. (2013). Platelet-rich plasma preparation for regenerative medicine: Optimization and quantification of cytokines and growth factors. Stem Cell Res. Ther..

[6] Woodall J., Tucci M., Mishra A., Asfour A., Benghuzzi H. (2008). Cellular effects of platelet rich plasmainterleukin1 release from prp treated macrophages. Biomed. Sci. Instrum..

[7] Piccin A., Di Pierro A.M., Canzian L., Primerano M., Corvetta D., Negri G., Mazzoleni G., Gastl G., Steurer M., Gentilini I. (2017). Platelet gel: A new therapeutic tool with great potential. Blood Transfus..

[8] (1988). The American Fertility Society classifications of adnexal adhesions, distal tubal occlusion, tubal occlusion secondary to tubal ligation, tubal pregnancies, müllerian anomalies and intrauterine adhesions. Fertil. Steril..

[9] Chang Y., Li J., Chen Y., Wei L., Yang X., Shi Y., Liang X. (2015). Autologous platelet-rich plasma promotes endometrial growth and improves pregnancy outcome during in vitro fertilization. Int. J. Clin. Exp. Med..

[10] Kusumi M., Ihana T., Kurosawa T., Ohashi Y., Tsutsumi O. (2020). Intrauterine administration of platelet-rich plasma improves embryo implantation by increasing the endometrial thickness in women with repeated implantation failure: A single-arm self-controlled trial. Reprod. Med. Biol..

[11] Gupta V., Parihar A.S., Pathak M., Sharma V.K. (2020). Comparison of Platelet-Rich Plasma Prepared Using Two Methods: Manual Double Spin Method versus a Commercially Available Automated Device. Indian Dermatol. Online J..

[12] Srinivasan V.R., Rekha M., Edsor E., Raja S.P., Kumar T.D., Kalaiselvan S. (2020). Assessment of Quality of Platelet-Rich Plasma Produced With Desktop Centrifuge and Comparison With Standardized Commercially Available Platelet-rich plasma. J. Pharm. Bioallied Sci..

[13] Foster T.E., Puskas B.L., Mandelbaum B.R., Gerhardt M.B., Rodeo S.A. (2009). Platelet-rich plasma: From basic science to clinical applications. Am. J. Sports Med..

[14] Nazari L., Salehpour S., Hosseini S., Sheibani S., Hosseinirad H. (2021). The Effects of Autologous Platelet-Rich Plasma on Pregnancy Outcomes in Repeated Implantation Failure Patients Undergoing Frozen Embryo Transfer: A Randomized Controlled Trial. Reprod. Sci..

[15] Javaheri A., Kianfar K., Pourmasumi S., Eftekhar M. (2020). Platelet-rich plasma in the management of Asherman’s syndrome: An RCT. Int. J. Reprod. Biomed..

[16] Marini M.G., Perrini C., Esposti P., Corradetti B., Bizzaro D., Riccaboni P., Fantinato E., Urbani G., Gelati G., Cremonesi F. (2016). Effects of platelet-rich plasma in a model of bovine endometrial inflammation in vitro. Reprod. Biol. Endocrinol..

[17] Aghajanova L., Houshdaran S., Balayan S., Manvelyan E., Irwin J.C., Huddleston H.G., Giudice L.C. (2018). In vitro evidence that platelet-rich plasma stimulates cellular processes involved in endometrial regeneration. J. Assist. Reprod. Genet..

[18] Vishnyakova P., Artemova D., Elchaninov A., Efendieva Z., Apolikhina I., Sukhikh G., Fatkhudinov T. (2020). Effects of platelet-rich plasma on mesenchymal stem cells isolated from rat uterus. PeerJ.

[19] Zhang P., Li D., Yang Z., Xue P., Liu X. (2022). Nrf2/HO-1 pathway is involved the anti-inflammatory action of intrauterine infusion of platelet-rich plasma against lipopolysaccharides in endometritis. Immunopharmacol. Immunotoxicol..

[20] Jang H.Y., Myoung S.M., Choe J.M., Kim T., Cheon Y.P., Kim Y.M., Park H. (2017). Effects of Autologous Platelet-Rich Plasma on Regeneration of Damaged Endometrium in Female Rats. Yonsei Med. J..

[21] Zhang S., Li P., Yuan Z., Tan J. (2019). Platelet-rich plasma improves therapeutic effects of menstrual blood-derived stromal cells in rat model of intrauterine adhesion. Stem Cell Res. Ther..

[22] Farimani M., Poorolajal J., Rabiee S., Bahmanzadeh M. (2017). Successful pregnancy and live birth after intrauterine administration of autologous platelet-rich plasma in a woman with recurrent implantation failure: A case report. Int. J. Reprod. Biomed..

[23] Zadehmodarres S., Salehpour S., Saharkhiz N., Nazari L. (2017). Treatment of thin endometrium with autologous platelet-rich plasma: A pilot study. JBRA Assist. Reprod..

[24] Eftekhar M., Neghab N., Naghshineh E., Khani P. (2018). Can autologous platelet rich plasma expand endometrial thickness and improve pregnancy rate during frozen-thawed embryo transfer cycle? A randomized clinical trial. Taiwan. J. Obstet. Gynecol..

[25] Aghajanova L., Cedars M.I., Huddleston H.G. (2018). Platelet-rich plasma in the management of Asherman syndrome: Case report. J. Assist. Reprod. Genet..

[26] Mohamed Amer M.I., Ahmed M.E.-S., Mokhtar Kamal R., Torky A.M.M.A.E. (2018). The Value of Using Platelet Rich Plasma after Hysteroscopic Analysis of Severe Intrauterine Adhesions (A Randomized Controlled Trial). Egypt. J. Hosp. Med..

[27] Peng J., Li M., Zeng H., Zeng Z., Huang J., Liang X. (2020). Intrauterine infusion of platelet-rich plasma is a treatment method for patients with intrauterine adhesions after hysteroscopy. Int. J. Gynaecol. Obstet. Off. Organ Int. Fed. Gynaecol. Obstet..

[28] Shen M., Duan H., Lv R., Lv C. (2022). Efficacy of autologous platelet-rich plasma in preventing adhesion reformation following hysteroscopic adhesiolysis: A randomized controlled trial. Reprod. Biomed. Online.

[29] Agarwal M., Mettler L., Jain S., Meshram S., Günther V., Alkatout I. (2020). Management of a Thin Endometrium by Hysteroscopic Instillation of Platelet-Rich Plasma into The Endomyometrial Junction: A Pilot Study. J. Clin. Med..

[30] Euser A.M., Zoccali C., Jager K.J., Dekker F.W. (2009). Cohort studies: Prospective versus retrospective. Nephron. Clin. Pract..

